# Correction: Electrospun ZIF-67/PVDF composite membranes for efficient ciprofloxacin removal from wastewater

**DOI:** 10.1039/d5ra90067k

**Published:** 2025-05-30

**Authors:** Ping Li, Xiaolin Yue, Anhong Li, Can Cui, Lan Wang, Wenyuan Tan

**Affiliations:** a College of Chemical Engineering, Sichuan University of Science and Engneering Zigong Sichuan China twyhyx@126.com; b College of Chemical and Environmental Engineering, Sichuan University of Science and Engineering Zigong Sichuan China

## Abstract

Correction for ‘Electrospun ZIF-67/PVDF composite membranes for efficient ciprofloxacin removal from wastewater’ by Ping Li *et al.*, *RSC Adv.*, 2025, **15**, 11503–11510, https://doi.org/10.1039/D5RA00237K.

The authors regret that the molecular weight of PVDF was not correctly given in the article. For the sentence beginning “Polyvinylidene fluoride (PVDF, Sinopharm, 400 000) particles...” on page 11504, the corrected sentence should read as follows: “Polyvinylidene fluoride (PVDF, Sinopharm, 1 000 000) particles, hexahydrate cobalt nitrate (Co(NO_3_)_2_·6H_2_O, Aladdin, 99.9%), methanol (Macklin, 99.5%), 2-methyl imidazole (Aladdin, 99%), polyvinyl pyrrolidone (Aladdin, K30), *N*,*N*-dimethylformamide (DMF, Aladdin, 99.5%), and ciprofloxacin (Aladdin, 98%) were of analytical grade and used without further purification”.

The Royal Society of Chemistry apologises for these errors and any consequent inconvenience to authors and readers.

